# Supplementary material for: Stenting versus balloon angioplasty alone in patients with below-the-knee disease: A propensity score-matched analysis
Source: PLoS One. 2021 Jun 10;16(6):e0251755. doi: 10.1371/journal.pone.0251755 (PMC8191955; doi:10.1371/journal.pone.0251755)
Supplement: Attachment — Submitted filename: Response-to-reviewer-PLOSONE.docx [file pone.0251755.s003.docx]

**RE:  PONE-D-21-00154
Stenting versus Balloon Angioplasty Alone in Patients with Below-the-Knee Disease: A Propensity Score-Matched Analysis
PLOS ONE**

**We have tried our best to make revisions to improve the quality of the manuscript to editor comment.**

**To make it easier for you to follow how changes were made, we have revised manuscript with red or blue color.**

**Please consider the following the authors’ response and revisions according to reviewer’ comments.**

**Thank you for your advice.**

**Journal Requirements:**

**When submitting your revision, we need you to address these additional requirements.**

1. **Please ensure that your manuscript meets PLOS ONE's style requirements, including those for file naming. The PLOS ONE style templates can be found at**

**https://journals.plos.org/plosone/s/file?id=wjVg/PLOSOne_formatting_sample_main_body.pdf and**

**https://journals.plos.org/plosone/s/file?id=ba62/PLOSOne_formatting_sample_title_authors_affiliations.pdf**

****Response**

**Thank you for your kind comment. We checked and revised the manuscript according to PLOS ONE's style requirements, including those for file naming**

**2. Thank you for stating the following financial disclosure:**

**No**

**At this time, please address the following queries:**

**2a)           Please clarify the sources of funding (financial or material support) for your study. List the grants or organizations that supported your study, including funding received from your institution.**

**2b)           State what role the funders took in the study. If the funders had no role in your study, please state: “The funders had no role in study design, data collection and analysis, decision to publish, or preparation of the manuscript.”**

**2c)           If any authors received a salary from any of your funders, please state which authors and which funders.**

**2d)           If you did not receive any funding for this study, please state: “The authors received no specific funding for this work.”**

**Please include your amended statements within your cover letter; we will change the online submission form on your behalf.**

****Response**

**Thanks for your kind comment. We put the sentece about stating the finacial disclosure into cover letter (“The authors received no specific funding for this work.”)**

**3. Thank you for stating the following in your Competing Interests section:**

**NO**

**Please complete your Competing Interests on the online submission form to state any Competing Interests. If you have no competing interests, please state "The authors have declared that no competing interests exist.", as detailed online in our guide for authors at http://journals.plos.org/plosone/s/submit-now**

**This information should be included in your cover letter; we will change the online submission form on your behalf.**

**Please know it is PLOS ONE policy for corresponding authors to declare, on behalf of all authors, all potential competing interests for the purposes of transparency. PLOS defines a competing interest as anything that interferes with, or could reasonably be perceived as interfering with, the full and objective presentation, peer review, editorial decision-making, or publication of research or non-research articles submitted to one of the journals. Competing interests can be financial or non-financial, professional, or personal. Competing interests can arise in relationship to an organization or another person. Please follow this link to our website for more details on competing interests:** [**http://journals.plos.org/plosone/s/competing-interests**](http://journals.plos.org/plosone/s/competing-interests)

****Response**

**Thanks for your kind comment. We put and add the sentece about state any competing interests section into cover letter. ("The authors have declared that no competing interests exist.")**

**4, Please amend your list of authors on the manuscript to ensure that each author is linked to an affiliation. Authors’ affiliations should reflect the institution where the work was done (if authors moved subsequently, you can also list the new affiliation stating “current affiliation:….” as necessary).**

****Response**

**Thanks for your kind comment. We already wrote and described the title page according to the authors’ current affiliation.**

**We have tried our best to make revisions to improve the quality of the manuscript to editor comment.**

**Please consider the following the authors’ response and revisions according to reviewer’ comments.**

**Thank you for your advice.**

**Reviewers' comments:

Reviewer's Responses to Questions**

**Comments to the Author

1. Is the manuscript technically sound, and do the data support the conclusions?

The manuscript must describe a technically sound piece of scientific research with data that supports the conclusions. Experiments must have been conducted rigorously, with appropriate controls, replication, and sample sizes. The conclusions must be drawn appropriately based on the data presented.**

**Reviewer #1: Yes**

**Reviewer #2: Yes**

**2. Has the statistical analysis been performed appropriately and rigorously?**

**Reviewer #1: Yes**

**Reviewer #2: Yes**

**3. Have the authors made all data underlying the findings in their manuscript fully available?

The** [**PLOS Data policy**](http://www.plosone.org/static/policies.action#sharing) **requires authors to make all data underlying the findings described in their manuscript fully available without restriction, with rare exception (please refer to the Data Availability Statement in the manuscript PDF file). The data should be provided as part of the manuscript or its supporting information, or deposited to a public repository. For example, in addition to summary statistics, the data points behind means, medians and variance measures should be available. If there are restrictions on publicly sharing data—e.g. participant privacy or use of data from a third party—those must be specified.**

**Reviewer #1: Yes**

**Reviewer #2: Yes**

**4. Is the manuscript presented in an intelligible fashion and written in standard English?

PLOS ONE does not copyedit accepted manuscripts, so the language in submitted articles must be clear, correct, and unambiguous. Any typographical or grammatical errors should be corrected at revision, so please note any specific errors here.**

**Reviewer #1: Yes**

**Reviewer #2: Yes**

**5. Review Comments to the Author

Please use the space provided to explain your answers to the questions above. You may also include additional comments for the author, including concerns about dual publication, research ethics, or publication ethics. (Please upload your review as an attachment if it exceeds 20,000 characters)**

**Reviewer #1: The authors here did the propensity matched analysis of stenting versus balloon angioplasty in the critical limb ischemia (CLI) patients with below the knee disease. The optimal treatment of below the knee lesions either with balloon angioplasty or stenting remains controversial. The data is sparse with majority of studies with small sample size.
The rationale and careful nature of the data review and statistical methods appear reasonably sound. The study limitations have been pointed out correctly. The authors showed no difference in cardiovascular clinical outcomes and target lesion related 1 year clinical outcomes in balloon angioplasty versus stenting group after propensity matching.

- Please correct second last line on page 4, “believe” has been used twice.**

****Response**

**Thanks for your kind comment. We changed and revised senteces as follows;**

**We believe the armamentarium of BMS compared to conventional balloons, particularly in bail-out situations, and believe that we can prove the safety and efficacy of BMS in the treatment of BTK lesions in a relatively large number of patients. This study aimed to retrospectively compare the safety and efficacy of stenting (bail-out or planned as primary stenting) with standard POBA) in BTK lesions for up to 1 year.**

**🡪 *We believe the armamentarium of BMS compared to conventional balloons, particularly in bail-out situations.*** ***And, we also predict that we can prove the safety and efficacy of BMS in the treatment of BTK lesions in a relatively large number of patients. This study aimed to retrospectively compare the safety and efficacy of stenting (bail-out or planned as primary stenting) with standard POBA in BTK lesions for up to 1 year.***

**Reviewer #2: This study aimed to evaluate the safety and efficacy of BMS compared to PTA in BTK lesions. It is a retrospective study that included 350+ patients that underwent either PTA or BMS for BTK. Propensity matching was performed to achieve matched groups. Both angiographic and clinical end points were analyzed. The authors conclude bare metal stents were similar in outcomes compared to PTA alone in these patients.

My comments are as follows:
1. The Cochrane review on this topic (Cochrane Database Syst Rev. 2018 Dec 8;12(12):CD009195) should be included either in the introduction section or discussion. This also concluded there is no significant added benefit of stenting compared to PTA alone for BTK.**

****Response**

**Thanks for your kind comment. We checked and put the sentences into introduction as follows;**

**Previous research related to BTK intervention comparing plain old balloon angioplasty (POBA) with stent implantation has been plagued by relatively small sample sizes, which might have affected the study results [11-14].**

**🡪 Previous research related to BTK intervention comparing plain old balloon angioplasty (POBA) with stent implantation has been plagued by relatively small sample sizes, which might have affected the study results [11-14]. *In addition, despite the accumulation of well-designed randomized comparative studies on stent implantation and POBA in the treatment of CLI, high level of evidence is still limited (***[15].

**2. The authors report the stent group was divided into elective or bailout stenting, although I do not see this difference reported in the results section. Were the results similar?**

****Response**

**Thanks for your kind comment. In this study, stent group includes both primary and bail-out stenting. A subgroup analysis of the comparison of primary and provisional stenting was not performed. After collection of more datas, we plan to derive the results of a multicenter randomized study on the comparison of primary stenting and provisional stenting. So we revised and changed sentences in ABSTRACT part as follows;**

**The stenting was decided either by primary stenting or by bail out stenting.**

**🡪** ***Stent group includes both primary and provisional stenting.***

**And delete the contents of the blanket in conclusion part 🡪 *~~(provisional and primary)~~***

**3. In the results section, page 11, it is reported that after PSM analysis, three matched groups of lesions were obtained. Yet in table 3 only 2 groups are reported. This needs clarification.**

****Response**

**Thanks for your kind comment**

**Thanks for your advise and comment. We change and revised sentence in result section. From “ three matched groups” to “ two matched groups”.**

**In the present study, a total of 654 lesions (stent lesion: n = 127, POBA lesion n = 527 lesion) were treated. After PSM analysis, three propensity-matched groups (stent lesion: n = 116, POBA lesion n = 329 lesion) were generated (Table 3).**

***🡪*** ***In the present study, a total of 654 lesions (stent lesion: n = 127, POBA lesion n = 527 lesion) were treated. After PSM analysis, two propensity-matched groups (stent lesion: n = 116, POBA lesion n = 329 lesion) were generated (Table 3).***

**4. In table 3, both columns read "all patients" shouldn't one of them be PSM group?**

****Response**

**Thanks for your comment. We changed one of “All Patients” to *“Matched Patients”* in table 3.**

**5. Why were clinical outcomes studied as end point? How would stenting versus PTA alone for BTK affect MACCE?**

****Response**

**Thnks for your kind comment. A lot of studies about interventional treatment of CLI used MACCE including myocardial infarction as the study point. Studies on the association between use of drug-coated balloons in thet treatment of peripheral arterial disease and late mortality have been reported, (J Am Heart Assoc. 2018;7:e011245. DOI: 10.1161/JAHA.118.011245.), and studies on use ofantiplatelet agents and clinical outcomes have also been reported** **J Clin Med 2020 Oct 30;9(11):3515.doi: 10.3390/jcm9113515). As reported previously, CLI itself is not just associated with MI, cardiovascular event and MACCE but also amputation and limb salavage is associated with clinical cardiovascular outcomes ( J Am Coll Cardiol. 2014 February 25; 63(7): 682–690. doi:10.1016/j.jacc.2013.09.073). In the literature of Wojciech Scczeklik et al., one of possible explanations for high risk for MACE is that revascularization procedures may result in myocardial injury at the time of intervention that may impact future prognosis (Clinical Research in Cardiology (2018) 107:319–328). So we studied and used clinical outcomes as a study end point including limb salvage and MACCE.**

**6. The authors conclude that stenting is comparable to PTA alone for BTK in regards to angiographic end points (re-stenosis), yet it should be stressed that despite higher acute luminal gain and less percentage residual stenosis with stents, the repeat revascularization rates were similar or higher in stent group. This to me should be interpreted as PTA appears to be sufficient for these lesions, with stents to be used in very select group of patients. Not the other way around.**

****Response**

**Thanks for your kind and detailed comments. We agree with your opinion that stents implantation could be used in very select groups. So we added sentences in discussion part as follows;**

**“In the present study, the 1-year clinical outcomes between the two groups were not significantly different in terms of amputation rate and cardiovascular clinical outcomes, including mortality. In addition, our data showed a higher trend in the incidence of TER in the stenting group than in the POBA group, but the results were not statistically significant after propensity matching.”**

**🡪** “**In the present study, the 1-year clinical outcomes between the two groups were not significantly different in terms of amputation rate and cardiovascular clinical outcomes, including mortality. In addition, our data showed a higher trend in the incidence of TER in the stenting group than in the POBA group, but the results were not statistically significant after propensity matching. *In addition, a numerically higher limb salvage rate was shown in stent group compared to POBA group, so stent implantation may remain a treatment option in special circumstances, such as flow limiting dissection and persistent recoil despite of repeated balloon dilatation.”***

**Also, we highligtened in conclusions part already as follows;**

**Although there was a trend toward a higher incidence of TER risk in the stenting group, stent implantation seemed to have acceptable 1-year safety and efficacy compared with POBA alone in CLI patients undergoing BTK PTA. These results indicate that stent implantation could remain the preferred strategy in special circumstances, such as flow-limiting dissection and recoil despite frequent prolonged balloon dilatation. Long-term randomized studies with larger study populations and unified methodologies are necessary to elucidate the final conclusion.**

**And we thought carefully, in the interpretation of the result value, the conclusion could be reached little differently. The results of our study showed no differences of clinical outcomes in both groups, especially amputation rates despite numerical differences of target extremity revascularization. So we carefully concluded the results about the role and necessity of BMS implantation in the interventional treatment of CLI.**

**6. PLOS authors have the option to publish the peer review history of their article (**[**what does this mean?**](https://journals.plos.org/plosone/s/editorial-and-peer-review-process#loc-peer-review-history)**). If published, this will include your full peer review and any attached files.**

****Response**

**Yes, I don’t care about open of review history.

If you choose “no”, your identity will remain anonymous but your review may still be made public.


Do you want your identity to be public for this peer review? For information about this choice, including consent withdrawal, please see our** [**Privacy Policy**](https://www.plos.org/privacy-policy)**.**

**Reviewer #1: Yes: Sukhdeep Bhogal**

**Reviewer #2: Yes: Madhan Sundaram**
